# Failure to detect *Plasmodium vivax *in West and Central Africa by PCR species typing

**DOI:** 10.1186/1475-2875-7-174

**Published:** 2008-09-11

**Authors:** Richard L Culleton, Toshihiro Mita, Mathieu Ndounga, Holger Unger, Pedro VL Cravo, Giacomo M Paganotti, Nobuyuki Takahashi, Akira Kaneko, Hideaki Eto, Halidou Tinto, Corine Karema, Umberto D'Alessandro, Virgilio do Rosário, Takatoshi Kobayakawa, Francine Ntoumi, Richard Carter, Kazuyuki Tanabe

**Affiliations:** 1 Laboratory of Malariology, International Research Centre of Infectious Diseases, Research Institute of Microbial Diseases, Osaka University, Osaka, Japan; 2 Tokyo Women's Medical University, Tokyo, Japan; 3 Centre d'Etudes des Resources Vegetales, Brazzaville, Republic of Congo; 4 Institute of Immunology and Infection Research, University of Edinburgh, Edinburgh, UK; 5 Centro de Malária e Outras Doenças Tropicais, Lisbon, Portugal; 6 Department of Medicine, Karolinska University Hospital, Solna, Sweden; 7 Istituto Pasteur, Fondazione Cenci-Bolognetti, Università di Roma 'La Sapienza', Rome, Italy; 8 Medical Research Unit, Albert Schweitzer Hospital, Lambaréné, Gabon; 9 Institute of Tropical Medicine, Antwerp, Belgium; 10 Centre Muraz, Bobo Dioulasso, Burkina Faso; 11 Programme Nationale de Lutte Integrée contre le Paludisme, Kigali, Rwanda; 12 Department of Protozoology, Institute of Tropical Medicine, Nagasaki University, Nagasaki, Japan

## Abstract

**Background:**

*Plasmodium vivax *is estimated to affect 75 million people annually. It is reportedly absent, however, from west and central Africa due to the high prevalence of the Duffy negative phenotype in the indigenous populations. Despite this, non-African travellers consistently return to their own countries with *P. vivax *malaria after visiting this region. An attempt was made, therefore, to detect the presence of *P. vivax *parasites in blood samples collected from the indigenous populations of west and central Africa.

**Methods:**

Parasite species typing (for all four human malaria parasites) was carried out by PCR on 2,588 blood samples collected from individuals from nine African malaria-endemic countries.

**Results:**

Most infections (98.5%) were *Plasmodium falciparum*, *Plasmodium malariae *was identified in 8.5% of all infections, and *Plasmodium ovale *in 3.9%. The prevalence of both parasites varied greatly by country. Only one case of *P. vivax *was detected from Sao Tome, an island off the west coast of Africa, confirming the scarcity of this parasite in Africa.

**Conclusion:**

The prevalence of *P. vivax *in local populations in sub-Saharan Africa is very low, despite the frequent identification of this parasite in non-African travellers.

## Background

*Plasmodium vivax *has the widest geographic range of the four parasites responsible for malaria in man. Historically, its range has extended as far north as Finland and northern China, and as far south as northern Australia and South Africa [1]. Concerted malaria control initiatives in countries in temperate zones have today confined *P. vivax *mainly to the tropics, where its range overlaps that of the most important malaria parasite in terms of public health, *Plasmodium falciparum*. Thus, the two parasites co-exist in large parts of the tropical and semi-tropical world, except, strikingly, in large parts of western and central Africa, where *P. vivax *appears to be almost completely absent [2]. This situation is apparently caused by the high prevalence of the Duffy negative phenotype in the local populations, which confers complete protection against *P. vivax *malaria [3]. The Duffy antigen/receptor for chemokines (DARC) is a transmembrane glycoprotein that is present on epithelial cells [4], endothelial cells [5], and erythrocytes. It is utilized by *P. vivax *parasites as the receptor for attachment to the red cell surface [6]. Duffy negative individuals are homozygous for a DARC allele, FY*B^*null*^, which carries a single nucleotide mutation which impairs promoter activity by disrupting a binding site for the h-GATA-1 erythroid transcription factor [7]. This results in the loss of DARC expression on erythrocytes, but does not affect expression in epithelial or endothelial cells. Individuals who are homozygous for this allele thus express no DARC protein on the red cell surface and are completely protected from the erythrocytic cycle of *P. vivax*. The Duffy negative phenotype occurs in over 95% of the population of west and central Africa, but is extremely rare outside Africa and the Arabian peninsula [8].

### Present day prevalence of *P. vivax *in Africa

Although *P. vivax *is known to be present in parts of northern, eastern and southern Africa, with some areas reporting a prevalence of around 20% of all malaria infections [9], it is extremely rare in west and central Africa. In fact, there are very few cases of *P. vivax *in the indigenous population at all, with the exception of the island of Sao Tome, which is known to harbour all four human malaria parasites [10]. It is possible that the lack of "local" *P. vivax *reported from these areas is due to the fact that its perceived absence precludes its identification. So ingrained may be the notion of the absence of *P. vivax *from west and central Africa that many surveys of parasite species composition from these areas do not include assays for the identification of the parasite [11], and many microscopists automatically designate any parasite associated with Schüffner's dotted erythrocytes as *P. ovale *[12]. There are, however, sporadic reports of its presence. One report from Equatorial Guinea describes the discovery of four cases of *P. vivax *in children of mixed race parentage [13], and another describes a mild infection of *P. vivax *in a Duffy negative woman from the Democratic Republic of Congo [14]. These rare accounts are supported by more extensive reports detailing *P. vivax *infections in non-African travellers returning from these areas. For example, an analysis of 618 imported *P. vivax *cases diagnosed in European clinics between 1999 and 2003, found that 17% of travellers had contracted the parasite in west and central Africa [15]. Furthermore, between 1995 and 1998 there were 73 reports of *P. vivax *imported into France from this region [16]. Imported malaria surveys from the USA report a similar pattern, with data from 2004 revealing that 65% of *P. vivax *imported into the USA from Africa in that year (n = 67) originated in countries in west and central regions [17]. As some of these reports [15,17] rely on microscopy for identification of parasite species, it may be argued that confusion between *P. ovale *and *P. vivax *may lead to a degree of misdiagnosis. However, there are an increasing number of reports that identify parasite species with molecular techniques [16,18], which are much less prone to misidentification of species. Given these data, it seems certain that transmission of *P. vivax *does occur in west and central Africa. However, it remains unclear how transmission is maintained in populations where the Duffy negative phenotype is almost at fixation.

In 1985, Van Ros described the presence of *P. vivax *in a Duffy negative individual [14] from the Democratic Republic of Congo. Recently, two new reports have also described this intriguing situation. One, from western Kenya, describes the presence of *P. vivax *circumsporozoite protein in 0.65% of mosquitoes from an area of high Duffy negativity [19]. More recently, Cavasini *et al *[20] reported clear evidence of *P. vivax *infections in two Duffy negative individuals in Brazil. It has been proposed that the parasite may be in the process of evolving mechanisms which allow the infection of Duffy negative individuals [21]. Such findings highlight the need for a clear investigation of the prevalence and population dynamics of *P. vivax *in west and central Africa, using accurate molecular species typing methods.

In order to assess the current prevalence of *P. vivax *in west and central Africa, PCR species typing of 2,588 samples from nine different countries throughout the continent was carried out.

## Methods

### Blood sample collection and parasite DNA extraction

**Figure 1:**
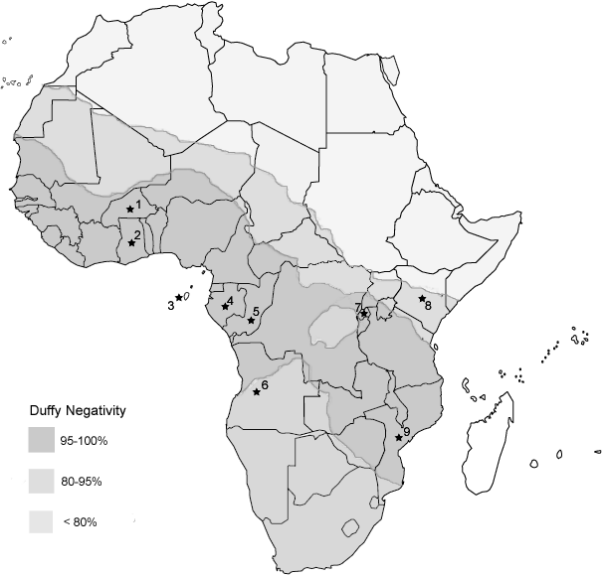


A total of 2,588 blood samples collated from various surveys undertaken in nine African countries (Figure 1) between 1998 and 2006 were analysed by PCR for the presence of each of the four human malaria parasites, *P. falciparum*, *P. vivax*, *P. malariae *and *P. ovale*. The original sample collections were predominantly carried out as part of unrelated investigations and the methodology of individual collection varies. Table 1 outlines the details of these collections. In all cases, ethical clearance for sampling was obtained from the relevant ethical committees (for samples collected specifically for this study, details are given in the following paragraphs).

**Table 1:** Details of sample collections.

**Collection area**	**Number**	**Sampling year**	Sampling method (ACD/PCD)*	**Age**	**Reference**
Burkina Faso^1^	108	2002	ACD	< 10	22
Republic of Congo	851	2005–2006	PCD (clinic)	all ages	This study
Gabon	206	2005	PCD (clinic)	all ages	This study
Ghana^1^	352	2004	ACD	< 15	This study
Kenya	722	1998	ACD	All ages	This study
Sao Tome^1,2^	70	2004	PCD (clinic)	All ages	23
Angola^1,2^	90	2003–2004	PCD (clinic)	1–5	24
Mozambique^1,2^	90	2004	PCD (clinic)	1–5	25
Rwanda^1,2^	99	2003	PCD (clinic)	All ages	26

Total	2588				

^1 ^Only *P. falciparum *positive samples were available for analysis

^2 ^Only *P. falciparum *single species infections (diagnosed by microscopy) were analysed

*ACD; active case detection, PCD; passive case detection

### Samples from Burkina Faso

In 2002, samples were collected from asymptomatic children under 10 years old by Active Case Detection (ACD) in two villages (Bassy and Zanga) 60 Km east of Ouagadougou, the capital of Burkina Faso at the end of the transmission season [22]. DNA was extracted by the use of TRIZOL^® ^reagent [22]. 108 *P. falciparum *positive samples (by PCR), were used in this study.

### Samples from Angola, Mozambique, Rwanda, and the Democratic Republic of Sao Tome & Principe (DRSTP)

Blood samples were collected by Passive Case Detection (PCD) at clinics in the following countries: DRSTP, February 2004, at the Centro Policlínico de Saúde de Água Grande, São Tomé (all ages)[23]; Angola, 2003–2004 at the Hospital Pediatrico de Luanda (1–5 year olds)[24]; Mozambique from July to October 2004 at the Hospital Central de Maputo (1–5 year olds)[25]; Rwanda, November to December 2003, at the Rukara Health Centre (1–60 years old)[26]. Only samples identified by thick and thin smear microscopy as *P. falciparum *single species infections were available for analysis. Blood samples were spotted onto Whatman^® ^n°4 (n°3 in the case of Rwanda) filter paper and parasite genomic DNA was obtained by boiling in Chelex-100 [27] and subsequent ethanol precipitation.

### Samples from Gabon

206 samples were collected specifically for this study. Samples were collected in and around Lambarene, Departement du Moyen Ogooué at five different locations (Hôpital Albert Schweitzer, Hôpital General de Lambaréné, Dispensaire d'Isaac, Adouma and PK48, a village 48 kilometres from Lambaréné). 100–200 μl of venous blood was applied to Whatman^® ^FTA^® ^Classic Filter paper cards (Whatman^®^, USA) and left to air dry. Whatman^® ^FTA^® ^filter cards deactivate viral DNA/RNA and preserve human and parasite DNA for downstream analyses. DNA extractions were carried out according to the manufacturer's instructions. Briefly, a disc of 1.2 mm in diameter was punched from the centre of each dried blood spotted card and washed three times with Whatman^® ^FTA^® ^Purification Reagent, and twice with TE buffer. This treated disc was then used directly in subsequent PCR analyses. Ethical clearance for sampling in Gabon was obtained from the ethical committee of the International Foundation of the Albert-Schweitzer Hospital and Edinburgh University. Prior to sampling each patient was informed about the study, consent was obtained (in the case of children parents/guardians gave informed consent) and medical follow-up was provided if needed.

### Samples from the Republic of Congo

359 samples were collected by passive case detection from two health centres (Madibou and Tenrikyo) within Brazzaville, the capital of the Republic of Congo, in 2005. No age restrictions were applied to sampled individuals. These samples were collected on Whatman^® ^FTA^® ^filter paper, and processed as previously described. A further 492 samples were collected in 2006 by PCD from three separate locations within the Republic of Congo, 150 from Pointe-Noire, on the west coast of the country, a further 201 from the Tenrikyo health centre in Brazzaville, and 141 from Gamboma, a town in the east. These samples were collected on Whatman^® ^31ETCHR filter paper, and DNA extraction was performed using the EZ1 BioRobot™ (QIAGEN, Hilden, Germany) according to the manufacturer's instructions. Ethical approval for this collection was obtained from the ethical committee at Osaka University, and sampling was authorized by the administrative authority of the Ministry for Research and Ministry for Health in the Republic of Congo. Informed consent was obtained from individual patients, and antimalarial treatment was provided when appropriate.

### Samples from Kenya

722 samples were collected in 1998 by active case detection in the Kisii district of Kenya. All age groups were sampled. Blood was collected by finger-prick on Whatman^® ^31ETCHR filter paper, and DNA extracted by boiling in Chelex-100 [27] and subsequent ethanol precipitation. Ethical clearance for this collection was obtained from the Ministries of Health and Education in Kenya.

### Samples from Ghana

352 samples were collected by ACD from 0–15 year old children in four villages near Winneba, a western coastal region of the country. Finger-prick blood was collected on Whatman^® ^31ETCHR filter paper, and DNA extraction was performed using the EZ1 BioRobot™. This study was approved by the Ministry of Health/Ghana Health Service.

### Species typing PCR

DNA extracted from all samples was subjected to *Plasmodium *species typing PCR based on the nested PCR technique developed by Snounou *et al *[28] with some modifications. Oligonucleotide primers were identical to those previously described [28] but the PCR conditions were modified as follows: For the first round of PCR, 1 μl of extracted DNA was added to 14.85 μl of dH_2_0, 1.75 μl of each primer (rPLU5 and rPLU6 at 5 uM), 2.5 μl of AmpliTaq Gold^® ^10× PCR Buffer II, 2 μl of 25 mM MgCl_2 _solution, 1 μl of dNTP mixture (2.5 mM each) and 0.15 μl of AmpliTaq Gold^® ^in a 25 μl reaction. The following cycling conditions were applied using a GeneAmp^® ^PCR 9700 thermocycler (Applied Biosystems, USA): 95°C for 10 min, 30 cycles of 57°C for 1 min, 72°C for 1 min, 94°C for 1 min and a final extension step of 72°C for 4 min. 1 μl of the resulting PCR product was used for the second round of PCR, with an identical reaction mix to that described for the first round (using pairs of species specific primers FAL-1 and FAL-2, VIV-1 and VIV-2, MAL-1 and MAL-2, and OVA-1 and OVA-2), and with the following cycle conditions: 95°C for 10 min, 32 cycles of 94°C for 1 min, 65°C for 1 min, and a final extension step of 65°C for 5 min. The resulting PCR products were visualized on 2% agarose gels, with the presence or absence of a band with each species primer pair indicative of the presence or absence of that species in the initial sample.

### Sensitivity of species diagnosis PCR

Prior to commencement of PCR analysis of field samples, a pilot experiment was carried out to assess the sensitivity of the PCR conditions detailed above. This protocol consistently detected the presence of *P. vivax *in a dilution of genomic DNA that theoretically contained one copy of the parasite genome per μl (data not shown). Due to variation in DNA extraction technique between sample collections, a consistent volume of blood corresponding to the 1 μl of extracted genomic DNA used in the PCR cannot be given. However, it is estimated that no less than 0.5 μl of initial blood sample was used in each reaction. Therefore, the PCR detection method used in this investigation should detect *P. vivax *parasites in infections of as low as two parasites per μl of blood. Furthermore, microscopic evaluation of parasite presence was available for all samples, and these correlated well with PCR results. Although the very rare occurrence of a microscopically positive sample being found to be PCR negative did occur, the vast majority of discrepancies between microscopy and PCR diagnosis involved species misdiagnosis by microscopy, and the detection of parasite infections by PCR in microscopically negative samples, as is expected due to the greater sensitivity of the PCR technique.

### Duffy status profiling

An FY* allele-specific PCR [29] was used to determine the Duffy status of the individual from Sao Tome infected with *P. vivax*. Product amplification took place in a 50 μl volume reaction containing 5 μl of 10× PCR buffer, 4 μl of 25 mM MgCl2, 0.1 mM of each dNTP, 2 μl of the two 5 μM allele specific primers, 1 μl of the 5 μM control primers and 6 units of AmpliTaq Gold^® ^DNA polymerase (Applied Biosystems, USA). Amplification conditions were as follows; denaturation and activation of the AmpliTaqGold DNA polymerase at 96°C for 8 min, then 10 cycles of 94° for 20 s and 69°C for 1 min, leading to 25 cycles of 94°C for 20 s, 64°C for 30 s and 72°C for 1 min, followed by 5 cycles of 94°C for 20 s, 62°C for 30 s and 72°C for 1 min. Amplification of a 411 bp fragment of the ABO gene acted as the internal control for each reaction.

## Results and discussion

### Prevalence of *P. vivax *in sub-Saharan Africa

1,711 samples were positive for *P. falciparum *(1,526 single species infections, 51 with *P. ovale*, 129 with *P. malariae*, one with *P. vivax *and four with both *P. malariae *and *P. ovale*), 67 for *P. ovale *(12 single infections, 51 mixed with *P. falciparum*, and four triple infections with *P. falciparum *and *P. malariae*) 147 for *P. malariae *(14 single infections, 129 mixed with *P. falciparum*, and four triple infections with *P. ovale *and *P. falciparum*) and one for *P. vivax *(mixed infection with *P. falciparum*) (Table 2). The only *P. vivax *infected sample came from a Duffy positive individual from Sao Tome, an island off the west coast of Africa. No *P. vivax *from any other location within the continent was detected, confirming the scarcity of this parasite in Africa. When excluding samples from Rwanda, Mozambique, Angola and Sao Tome (self-selected as patients identified by microscopy with a mixed infection were excluded), *P. malariae *infections represented 8.5% of all malaria infections, and *P. ovale *3.9%. The prevalence of both parasites varies greatly by country.

**Table 2:** Species composition of isolates analysed by PCR (%)

**Collection area**	**Number**	***P. falciparum***	***P. ovale***	***P. malariae***	***P. vivax***
Burkina Faso^1^	108	108 (100)	6 (5.5)	8 (7.4)	0
Congo	851	341 (40.1)	11 (1.3)	8 (0.9)	0
Gabon	206	102 (49.5)	4 (1.9)	1 (0.5)	0
Ghana^1^	352	352 (100)	8 (2.3)	45 (12.8)	0
Kenya	722	459 (63.6)	35 (4.8)	84 (11.6)	0
Angola^1,2^	90	90 (100)	0	0	0
Mozambique^1,2^	90	90 (100)	0	0	0

Rwanda^1,2^	99	99 (100)	2 (2)	1 (1)	0
Sao Tome^1,2^	70	70 (100)	1 (1.4)	0	1 (1.4)

*Total*	*2588*	*1711*	*67*	*147*	*1*

^1 ^Only *P. falciparum *positive samples were available for analysis

^2 ^Only *P. falciparum *single species infections (diagnosed by microscopy) were analysed

### Scarcity of *P. vivax*

The prevalence of *P. vivax *in Africa is very low; no evidence for its presence in over 2,500 samples from nine African countries was found, with the exception of the island of Sao Tome, from which the parasite had previously been reported [10]. These results, combined with the sporadic reports of the transmission of *P. vivax *in indigenous populations [10,13,19] and the continued identification of imported cases originating in west and central Africa [15,17] indicate that a very low prevalence of *P. vivax *is sufficient to maintain transmission. It is conceivable that in areas with very high entomological inoculation rates (EIR), such as in many areas of west and central Africa [30], even very low numbers of Duffy positive individuals may allow the continued transmission of *P. vivax*.

That low numbers of Duffy positive individuals in west and central Africa are sufficient to maintain transmission on *P. vivax*, is not surprising considering the very high basic reproduction number of malaria in this region. The basic reproductive number of a pathogen, R_0_, is defined as the number of new infections arising from an infected individual introduced into a naïve population. When R_0 _> 1, transmission is maintained in a population, but when R_0 _< 1, transmission is interrupted and the pathogen cannot persist. A recent report showed that R_0 _for *P. falciparum *malaria transmission in Africa ranges from below one to nearly 11,000 with a median value of 86, depending on geographical location [30]. Given that both *P. falciparum *and *P. vivax *are vectored by the same mosquito species, the only factor that that differentiates R_0 _for both species in a given population is the human host's susceptibility to infection. In order for transmission to be blocked, the proportion of completely immune individuals (*p*) required in a population is given by the formula *p *> 1 - 1/R_0_. Thus, in areas where Duffy negativity is present in a population at a prevalence of up to 99%, as it is in many parts of west and Central Africa [8], then *P. vivax *transmission can be expected to occur when R_0 _(for *P. falciparum*) > 100, an entirely realistic value for many areas. It is entirely conceivable therefore, that *P. vivax *transmission occurs in populations in which there are a very high proportion Duffy negative individuals, given the very high *P. falciparum *R_0 _values associated with west and central Africa.

A number of researchers have recently suggested that *P. vivax *may be in the process of evolving mechanisms [21] that enable it to infect Duffy positive individuals. Given the extremely high EIRs and transmission dynamics of malaria parasites in sub-Saharan Africa, this scenario would appear highly unlikely given the extremely low incidences of the parasite reported here. Any *P. vivax *parasite that acquired the ability to infect Duffy negative individuals may be expected to rapidly spread throughout sub-Saharan Africa, and would be readily detectable in the population.

### Discrepancy between *P. vivax *rates in travellers and localpopulations

Surveillance of malaria cases imported into the USA between 2001 and 2005 [31-35], reveals that 32 cases of *P. vivax *originated in four west and central African countries for which we also have species prevalence data from the local populations. In the same time period, there were 545 cases of imported *P. falciparum *from the same countries. This gives a ratio of 100:6 *P. falciparum *to *P. vivax *infections in these areas, a surprisingly high rate, especially considering that *P. malariae *and *P. ovale *are represented at ratios of 100:6 and 100:5 respectively (comparable with those of the local populations, see Table 3). How does one account, then, for the discrepancy between the imported *P. vivax *data, and the extremely low prevalence in the native population reported here?

**Table 3:** Parasite species prevalence of traveler's malaria imported into the USA (2001-2005) from Burkina Faso, Gabon, Republic of Congo / Democratic Republic of Congo and Ghana compared to that of the local populations.

**Species**	**Total number**		**Species prevalence (per 100 *P. falciparum *cases)**	
	Imported to the USA^1^	Local population^2^	Imported to the USA^1^	Local population^2^

*P. falciparum*	545	903	-	-
*P. vivax*	32	0	**5.9**	**0**
*P. malariae*	34	62	6.2	6.9
*P. ovale*	26	29	4.8	3.2

^1 ^2001–2005, data from [17,31-35]

^2 ^data from the current survey

It is possible that the geographical distribution of *P. vivax *within Africa is patchy, with sporadic areas of transmission scattered throughout the continent, possibly associated with human populations in which the Duffy negative phenotype is present at a lower frequency than elsewhere (such as on the island of Sao Tome). Travellers may preferentially visit areas of west and central Africa where there is a relatively high frequency of Duffy positive individuals in the local population (*e.g*. migrant workers and non-African expatriates) and where *P. vivax *is more likely to be transmitted.

Another factor that may contribute to this discrepancy is the higher transmissibility of *P. vivax *relative to other malaria parasites, and in particular relative to *P. falciparum*, under adverse conditions [9]. This has the consequence that there should be higher proportion of *P. vivax *relative to *P. falciparum *in the vector mosquitoes than there is in the corresponding human population. Consequently travellers, who are a probe of the infection rates in the local mosquitoes, can be expected to, and indeed do have (Carter and Mendis, unpublished analysis), higher proportions of *P. vivax *than are found in the endemic human populations amongst whom the travellers have briefly resided. This may explain the recent findings of Ryan et al [19], who report the presence of *P. vivax *in 0.65% of mosquitoes from an area of western Kenya with a high proportion of Duffy negativity in the local population. However, even an extremely small percentage of Duffy positive individuals in this population may be expected to support such a rate in mosquitoes.

The use of prophylactic anti-malaria drugs among travellers may also contribute to this phenomenon. Mefloquine is the recommended prophylactic drug for travellers to west and central Africa from the USA [31], and whilst effective against the blood stages of all malaria parasites, it does not affect the dormant hypnozoite stages of *P. vivax*, and will therefore not protect against relapses after cessation of drug use. This is also true of *P. ovale*, which is also capable of producing hypnozoites, and may explain the slightly higher rate of this parasite in returning travellers compared to the local populations (Table 3).

It is also probable that a small proportion of imported cases may be *P. ovale *infections rather than *P. vivax*, as is often difficult to distinguish the two species by microscopy [15]. As previously mentioned, there are an increasing number of reports detailing imported African *P. vivax *diagnosed by accurate molecular typing techniques [16,18].

In conclusion, the present study indicates that the prevalence of *P. vivax *in local populations in sub-Saharan Africa is very low, despite the frequent identification of this parasite in travellers. *P. vivax *malaria, therefore, does not constitute a health risk to the indigenous populations of west and central Africa, though Duffy positive individuals, including non-African travellers to the area, may be at risk.

## Competing interests

The authors declare that they have no competing interests.

## Authors' contributions

RCu, RCa and KT conceived the study and participated in its design and coordination. RCu wrote the manuscript. MN, FN, HU, RCa, UdA, AK and KT helped to draft the manuscript. RCu, HU, TM, HE and NT performed molecular parasite typing. MN coordinated and carried out sample collection in the Republic of Congo, HU and FN in Gabon, GP in Burkina Faso, HT, CK and UdA in Rwanda, PC and VdR in Sao Tome, Angola, and Mozambique, and TM, AK and TK in Ghana and Kenya. All authors read and approved the final manuscript.
